# Novel Targets for Treating Ischemia-Reperfusion Injury in the Liver

**DOI:** 10.3390/ijms19051302

**Published:** 2018-04-26

**Authors:** Weili Yang, Ji Chen, Yuhong Meng, Zhenzhen Chen, Jichun Yang

**Affiliations:** Department of Physiology and Pathophysiology, School of Basic Medical Sciences Key Laboratory of Molecular Cardiovascular Sciences of the Ministry of Education Center for Non-Coding RNA Medicine, Peking University Health Science Center, Beijing 100191, China; weiliyang0321@163.com (W.Y.); chenji72592cn@126.com (J.C.); mengyuhong@bjmu.edu.cn (Y.M.); chenzhenzhen@bjmu.edu.cn (Z.C.)

**Keywords:** liver ischemia-reperfusion injury, PPARγ, *FAM3A*, miRNA, LncRNA

## Abstract

Liver ischemia-reperfusion injury (IRI) is a major complication of hemorrhagic shock, liver transplantation, and other liver surgeries. It is one of the leading causes for post-surgery hepatic dysfunction, always leading to morbidity and mortality. Several strategies, such as low-temperature reperfusion and ischemic preconditioning, are useful for ameliorating liver IRI in animal models. However, these methods are difficult to perform in clinical surgeries. It has been reported that the activation of peroxisome proliferator activated receptor gamma (PPARγ) protects the liver against IRI, but with unidentified direct target gene(s) and unclear mechanism(s). Recently, *FAM3A*, a direct target gene of PPARγ, had been shown to mediate PPARγ’s protective effects in liver IRI. Moreover, noncoding RNAs, including LncRNAs and miRNAs, had also been reported to play important roles in the process of hepatic IRI. This review briefly discussed the roles and mechanisms of several classes of important molecules, including PPARγ, *FAM3A*, miRNAs, and LncRNAs, in liver IRI. In particular, oral administration of PPARγ agonists before liver surgery or liver transplantation to activate hepatic *FAM3A* pathways holds great promise for attenuating human liver IRI.

## 1. Introduction

Good blood circulation is the prerequisite for organs to maintain normal function. Ischemic diseases are common in clinic and have serious damages to organs. In some conditions, when blood flow is restored, the damages to ischemic organs will be exaggerated. This phenomenon is referred to as ischemia-reperfusion injury (IRI). Liver IRI sometimes occurs in liver transplantation, complex liver resection, hemorrhagic shock, and severe liver trauma surgery due to long ischemic time or other reasons [1]. IRI often leads to liver congestion, progressive thrombosis, and necrosis of organs, resulting in the failure of operation [2]. In western countries, about 30% of the general population suffers from hepatic steatosis, which has now become the most widespread cause of chronic liver disease. It has been realized that steatotic livers are more susceptible to injuries including IRI than healthy livers [3]. In the United States, liver transplantations account for approximately 23% of the total transplant surgeries [4], and many patients suffer from IRI. Only in 2016, about 8000 patients received liver transplants in the United States [5]. Hepatocellular carcinoma (HCC) is the third most common cause of cancer-related death worldwide [6]. In China, hepatocellular carcinoma (HCC) is one of the most common malignant tumors with poor prognosis [7]. Most of the HCC patients might need surgery and then suffer from liver IRI. Clearly, liver IRI has become a severe clinical issue. Generally, the pathophysiological process of liver IRI consists of acute and chronic phases. In the acute phase, injury of hepatocytes occurred in 3–6 h after reperfusion, accompanied by the overproduction of free radical and activation of T lymphocytes and macrophages. The chronic phase happened at 18–24 h after reperfusion with a large amount of neutrophil infiltration. The interactions among white blood cells, cytokines, and chemokines lead to increased neutrophils [8,9].

The mechanisms of liver IRI are complicated and have remained largely unclear. In the past decades, it had been revealed that mitochondrial dysfunction, reactive oxygen species (ROS) overproduction, calcium overload, activation of apoptotic kinases, proteases, and phospholipases play important roles in the pathogenesis of liver IRI [10]. Moreover, the nuclear factor kappa-light-chain-enhancer of activated B cells (NF-κB) signaling pathway and inflammatory process are also involved in liver IRI [11,12].

## 2. Current Strategies for Ameliorating Liver Ischemia-Reperfusion Injury (IRI)

Quickly restoring blood supply of ischemic liver as soon as possible is crucial for avoiding or reducing IRI. There are three stages in the progression of IRI: perfusion, ischemia, and reperfusion [13].

Some strategies, including ischemic preconditioning, ischemic postconditioning, and machine perfusion, are already used to attenuate the damage induced by reperfusion. During the period of the perfusion, a short-term ischemic preconditioning, to make the organism adaptive to the subsequent long ischemia, is an effective endogenous protective phenomenon, but the mechanism is poorly understood [14]. De Almeida et al. illuminated that hepatic preconditioning ameliorated liver IRI by reducing lipid peroxidation [15]. In addition, hypothermic preconditioning, remote ischemic preconditioning (RIPC), and pharmacological pretreatment with statins are a benefit to IRI [16,17,18]. Although ischemic preconditioning protects organs against IRI, the ischemic preconditioning time is very critical. In addition, the ischemic preconditioning is carried out prior to surgery, and is limited for treating acute liver injuries. The difficulty in properly determining the time and number of reperfusion/reocclusion cycles limits the clinical use of ischemic preconditioning [19]. In the stage of ischemia, the organ should be kept in a cold storage condition to reduce metabolism and organ dysfunction. Moreover, the machine perfusion, including hypothermic machine perfusion (HMP), hypothermic oxygenated perfusion (HOPE), and normothermic machine perfusion (NMP), can be useful for maintaining the physiological state and preserving the organ functions [20]. In the stage of reperfusion, ischemic postconditioning can also be used to ameliorate IRI [21]. Ischemic postconditioning alone or ischemic postconditioning combined with statin pretreatment could ameliorate reperfusion injuries [16,22]. However, application of ischemic postconditioning in tissue protection is also restricted due to the diversity in tissues’ physiological requirements [23].

Although these methods are effective in reducing liver IRI, they are expensive and sometimes hard to perform in clinical surgeries. Moreover, the current strategies are difficult in maintaining liver functions in the case of acute injuries. Activating certain key survival pathways and/or inhibit apoptotic/necrotic pathways by drugs or small molecules may be useful for reducing liver IRI before, or during, liver surgeries or transplantation. In the current review, we summarized and discussed the latest findings regarding the roles and mechanisms of peroxisome proliferator-activated receptor gamma (PPARγ), *FAM3A*, and noncoding RNAs in liver IRI. In particular, the potential of targeting certain important molecules, such as the PPARγ-*FAM3A* axis, to ameliorate liver IRI will be discussed.

## 3. PPARγ in Liver IRI

PPARγ is a member of the PPAR subfamily consists of PPARα, PPARβ/δ, and PPARγ [24,25]. PPARγ is initially reported to be highly expressed in adipose tissue. However, it is also widely expressed in many other tissues including livers [24,25]. After being bound by endogenous or synthetic ligands, PPARγ heterodimerizes with another nuclear receptor, retinoid X receptor alpha (RXRα). The heterodimer binds to the PPAR-response element (PPRE) in promoter regions of the target genes, and induces or represses gene transcription [26,27]. At present, the synthetic agonists of PPARγ, such as rosiglitazone and pioglitazone, are widely used for treating insulin resistance and type-2 diabetes [28].

More recently, it has been revealed that PPARγ also plays important roles in hepatic IRI [29]. In mouse liver after IRI, the expression and activity of PPARγ is increased, which protects hepatocytes against apoptosis and necrosis [30]. In steatotic livers, pharmacologic drugs that block angiotensin II actions could reduce the adverse effects of IRI by stimulating bradykinin (BK) generation. BK is further shown to exert its protective effects against hepatic injury via the activation of PPARγ [31]. Shin et al. reported that inhibition of PPARγ activity aggravated liver IRI [32]. Moreover, the authors further found that the activation of PPARγ during ischemia is age-dependent. PPARγ activation is lost after 30 min in adult and old mice, whereas it remained actively throughout ischemia in young mice [33]. Xu et al. reported that asiatic acid (AA) significantly attenuated hepatic IRI via the activation of PPARγ/NLRP3 inflammasome signaling pathway in mice [34]. Chen et al. reported that 15-Deoxy-Delta(12,14)-prostaglandin J2 (15d-PGJ2) pretreatment inhibited ROS generation and apoptosis in mouse livers after IRI. Moreover, these beneficial effects were partly reversed by antagonism of PPARγ [35]. Ruan et al. reported that limb remote ischemic preconditioning (RIPC) substantially activated PPARγ to exert the protective effects in liver IRI by the activation of autophagy [36]. Several other studies also suggested that PPARγ activation by TZDs, losartan or other molecules is beneficial for hepatocyte survival in liver IRI [7,37,38,39,40,41,42,43]. PPARγ deletion exaggerates liver IRI in animal models [32,44,45]. Collectively, PPARγ activation has been established to ameliorate liver IRI. Inhibition of inflammation and activation of autophagy had been proposed to explain the beneficial effects of PPARγ activation. However, the direct target gene(s) of PPARγ that mediates the beneficial effects of PPARγ activation in liver IRI still remains unclear [45], restricting the clinical use of PPARγ agonists in attenuating liver IRI in clinics.

## 4. *FAM3A* in PPARγ’s Protection in Liver IRI

Recently, *FAM3A*, the first member of the sequence similarity 3 (FAM3) gene family, has been identified as a novel target gene of PPARγ [46]. *FAM3A* is further shown to be a new mitochondrial protein, and it enhances ATP synthesis and release. Released ATP will activate P2 receptors to slightly increase cytosolic free calcium level and activate calmodulin (CaM), finally leading to the activation of the PI3K-Akt signaling pathway in hepatocytes [47,48]. *FAM3A* plays important roles in suppressing hepatic gluconeogenesis and lipogenesis via the activation of ATP-P2 receptor-Akt and adiponectin receptor-AMPK pathways [47,48]. Moreover, *FAM3A* also activates Akt pathways to induce the proliferation of vascular smooth muscle cells (VSMCs) and 3T3L1 preadipocytes [49,50]. Akt has been shown to function as an important survival kinase by phosphorylating a number of apoptotic molecules including bisphenol A disalicyate (BAD), forkhead transcription factors, caspase 9, forkhead box protein O1 (FOXO1), IkappaB kinase (IKK), and glycogen synthase kinase 3β (GSK-3β) beyond its well-known roles in regulating glucose and lipid metabolism [51,52,53]. Akt activation exerts beneficial effects in liver IRI [51,52,53]. Given that ATP production and Akt play crucial roles in the protection of liver IRI [53,54], it is reasonable to speculate that *FAM3A* may be involved in liver IRI process by directly stimulating ATP production to activate Akt pathways. Moreover, demonstrating whether *FAM3A* mediates the hepatoprotective effects of PPARγ in liver IRI is also of great significance.

Both PPARγ and *FAM3A* expression were upregulated in mouse liver after IRI [55]. Silencing of hepatic *FAM3A* markedly exaggerated liver IRI with repressed ATP production and Akt activity, reduced anti-apoptotic gene expression and increased pro-apoptotic gene expression in the livers. Pretreatment with rosiglitazone significantly ameliorated liver IRI with increased *FAM3A* expression, ATP production, and Akt activity in the liver [55].

**Figure 1:**
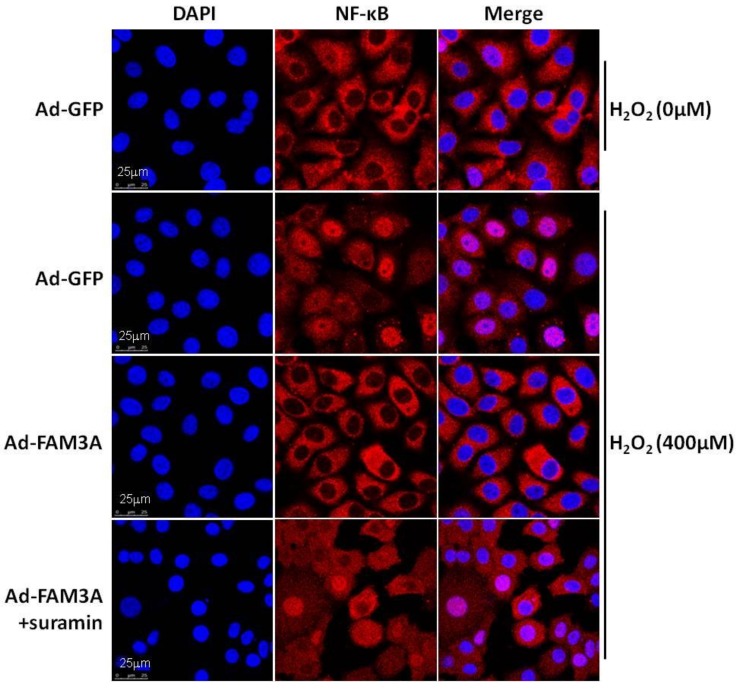
*FAM3A* inhibited NF-κB activation induced by oxidative stress in HepG2 cells. Oxidative stress increased nuclear localization of NF-κB, which was reversed by *FAM3A* overexpression. Moreover, *FAM3A*-induced inhibition of NF-κB was partially reversed by P2 receptor antagonist suramin. The scale bar indicated in the images is 25μm.

*FAM3A*-dificient mice exhibit normal liver functions and structure as wild-type (Wt) mice in physiological condition. However, *FAM3A*-dificient mice exhibited more severe liver damage after IRI with a reduction in hepatic ATP production and Akt activity when compared with Wt mice. Rosiglitazone pretreatment activated *FAM3A*-ATP-Akt pathway to protect against liver damage in Wt mice. However, rosiglitazone failed to increase ATP content and activate the Akt pathways in *FAM3A*-deificient mouse livers. Rosiglitazone pretreatment also failed to protect liver against IRI after *FAM3A* knockout [55]. In cultured hepatocytes, rosiglitazone-induced Akt activation is also dependent on *FAM3A*-ATP pathway. Beyond Akt survival pathways, NF-κB also plays important roles in liver IRI by regulating the expression of proinflammatory cytokines [56]. *FAM3A* repressed NF-κB activity and inflammation in IRI mouse livers [55]. Rosiglitazone pretreatment significantly repressed NF-κB activity and proinflammatory cytokine expression in Wt mouse livers, but not in *FAM3A*-deificient mouse livers [55]. Moreover, *FAM3A*’s inhibition on NF-κB activation was partially repressed by P2 receptor antagonist, revealing the involvement of ATP-P2 receptor pathway in *FAM3A’*s inhibition on NF-κB activity in hepatocytes (Figure 1). Mice with hepatic *FAM3A* knockdown or knockout exhibited more severe oxidative stress in the circulation and liver after liver IRI. Rosiglitazone pretreatment significantly ameliorated global and hepatic oxidative stress in Wt mice but not in *FAM3A*-deficient mice [55]. In support, *FAM3A* also protects neuronal HT22 cells against apoptosis triggered by oxidative and endoplasmic reticulum (ER) stress [57,58].

**Figure 2:**
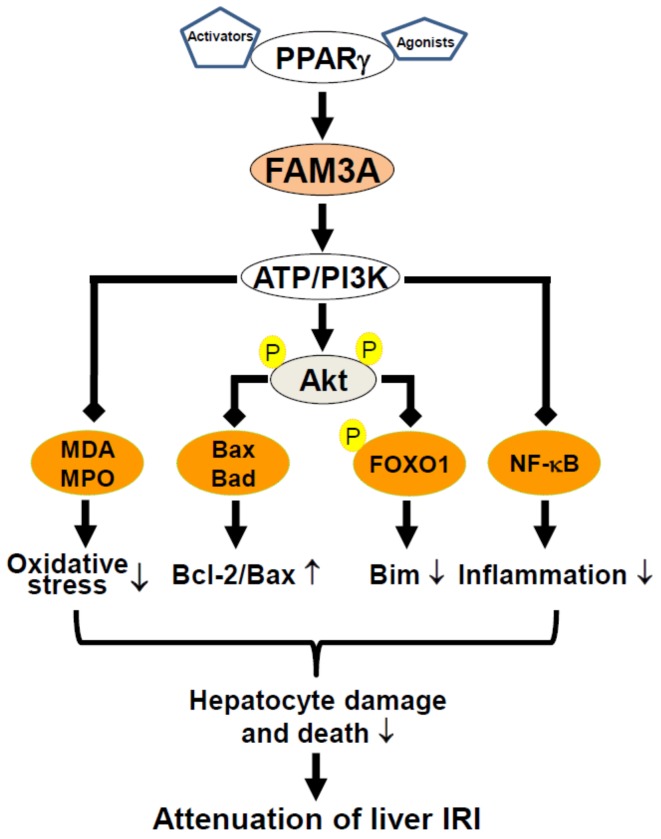
Proposed protective mechanisms of PPARγ-*FAM3A* axis in liver IRI. *FAM3A* exerts beneficial effects on liver IRI via the activation of ATP-PI3K-Akt pathways, inhibition of inflammation, and attenuation of oxidative stress. As a direct target gene of PPARγ, *FAM3A* mediates the protective effects of PPARγ activation on liver IRI. IRI, ischemia/reperfusion injury. MDA, methane dicarboxylic aldehyde; MPO, myeloperoxidase.

Collectively, *FAM3A* protects liver against IRI by activating Akt survival pathways, repressing NF-κB activation and attenuating oxidative stress. Importantly, the protective effects of PPARγ activation on liver IRI are achieved through the activation of *FAM3A* pathways. Ours and others findings strongly suggested that oral administration of PPARγ agonists before liver surgery or liver transplantation to activate hepatic *FAM3A* pathways is an effective strategy for attenuating human liver IRI [32,44,45,55] (Figure 2).

## 5. miRNAs and Liver IRI

Noncoding RNAs are classified into small noncoding RNAs (<200 bp) and long noncoding RNAs (LncRNAs, ≥200 bp) based on the length [59]. miRNAs are one class of small noncoding RNAs of 19–24 nucleotides that are the most important gene regulators at the post-transcriptional level [60,61,62]. So far, miRNAs have been reported to be tightly associated with many diseases [60,61,62]. Importantly, miRNAs are also involved in IRI processes of several organs, including the heart, kidney, brain, and liver. Ng, K.T. et al. [63] reported that several circulating miRNAs, including miR-148a, miR-1246, and miR-1290, were upregulated after liver transplantation. Zheng et al. [64] reported that global expression profiles of miRNAs in mouse livers were different between not only the reperfusion sample and the sham control, but also the ischemia sample and the sham control. Moreover, the miRNA profile was also deregulated in the livers of rats, mice, and humans after liver IRI [65,66,67]. In this review, the latest research regarding the roles and mechanisms of certain important miRNAs in liver IRI will be discussed.

### 5.1. miR-122 and Liver IRI

miR-122 is the most abundant miRNA in the liver. It has been reported that miR-122 is exclusively and abundantly expressed in hepatocytes, accounting for almost 70% of the total hepatic miRNAs [68,69]. miR-122 has been reported to play important roles in regulating hepatic glucose and lipid metabolism, and is tightly associated with the progression of HCC [70,71,72]. More recently, there had been increasing evidence that miR-122 was also involved in liver IRI. Andersson et al. [73] reported that miR-122 could be secreted into circulation in an ischemic porcine cardiogenic shock model. They found that circulating miR-122 level was increased by 460,000 folds after cardiogenic shock and significantly decreased after therapy. Roderburg et al. [74] reported that the circulating miR-122 level was correlated with liver injury in both mice and humans. Yang et al. [75] further showed that the circulating miR-122 level was significantly increased after 45 min of hepatic ischemia and 1–24 h of reperfusion. Moreover, the circulating miR-122 level is correlated with histological characteristics of necrosis and liver damage. Van Caster et al. [76] reported that the circulating miR-122 level was increased and correlated with the serum activities of aspartate transaminase (AST), alanine transaminase (ALT), and lactate dehydrogenase (LDH) after warm hepatic IRI. Selten et al. [77] demonstrated that the absolute miR-122 level and relative miR-122/miR-222 ratio in graft preservation solution are associated with early allograft dysfunction (EAD) and early graft loss after liver transplantation. Clearly, these findings revealed that deregulated miR-122 level in the circulation and liver is tightly associated with hepatocyte IRI.

Recently, the mechanisms of miR-122 in liver IRI have been determined. Xiao et al. [78] found that miR-122 was increased in an anoxia-reoxygenation injury model in L02 cells. They identified that mild hypothermia pretreatment could induce a significant downregulation in miR-122 expression and reduce the hepatocellular injury, and these effects was abrogated by the miR-122 mimic. The authors further discovered that miR-122 inhibition increased the expression of insulin-like growth factor-1 receptor (IGF-1R), one potential target gene of miR-122, which inhibited apoptosis via the IGF-1R/AKT signaling pathway in L02 cells. Another study by Akbari et al. [79] revealed that crocin, a water-soluble active component of saffron, protected the liver against IRI by increasing the activity of antioxidant enzymes. The author further showed that inhibition of miR-122 contributed to crocin’s protective effects on liver IRI. Mard et al. [80] also found that crocin, ZnSO_4_, and their combination, protected the liver against IRI by inhibiting miR-122 expression. Collectively, miR-122 serves as not only a novel biomarker, but also a potential therapeutic target for liver IRI. Inhibiting hepatic miR-122 expression is one potential method for ameliorating liver IRI.

### 5.2. miR-34a and Liver IRI

The mature miR-34 subfamily consists of three members: miR-34a, miR-34b, and miR-34c, respectively. miR-34a has been shown to directly inhibit the expression of p53 that inhibits cell proliferation and regulates liver function [79].

The gasotransmitter H_2_S protects several tissues, including the liver against IRI [81]. Huang et al. [81] determined the hepatoprotective effects of H_2_S on hepatic IRI in young (three months) and old rats (20 months). They found that NaHS administration ameliorated liver IRI in young rats, but not old ones, by activating the nuclear erythroid-related factor 2 (Nrf2) signaling pathway. Nrf2, a direct target gene of miR-34a, exerts antioxidative effects by regulating the expression of antioxidative genes, including glutathione S-transferase (GST) and SODs [81]. miR-34a upregulation impairs, while miR-34a downregulation enhances the hepatoprotective effect of H_2_S in the young and old rats through the modulation of the miR-34a/Nrf2 signaling pathway. Carbon monoxide (CO), a product of heme oxygenase (HO), also ameliorates IRI in various tissues thorough anti-inflammatory, vasodilatory, and anti-apoptotic effects with unclear mechanisms [82]. Kim et al. [82] reported that CO increased NAD-dependent deacetylase sirtuin-1 (SIRT1) expression by inhibiting miR-34a expression in livers after IRI. CO treatment inhibited miR-34a expression with increased SIRT1 expression in hepatocytes in the presence of oxidative stress, and rescued SIRT1 expression in miR-34a-transfected cells. Finally, an increase in SIRT1 expression protects against liver damage through p65/p53 deacetylation, which inhibits inflammatory responses and hepatocyte apoptosis. Carnosic acid (CA) has been reported to exert protective effect against organ injury by inhibiting apoptosis [83]. Shan et al. [83] reported that CA’s antiapoptotic effects in liver IRI was also achieved through the inhibition of miR-34a expression. Akbari et al. found that [79] crocin pretreatment also ameliorated liver IRI by inhibiting miR-34a expression. Moreover, Mard et al. [80] found that inhibition of miR-34a contributed to the protective effects of ZnSO_4_ and crocin cocktail in liver IRI. Collectively, miR-34a exerts deleterious effects in liver IRI by inhibiting the expression of Nrf2 and SIRT1.

### 5.3. miR-223 and Liver IRI

miR-223 was firstly identified as a regulator of hematopoietic linage differentiation. Recently, miR-223 was shown to be repressed in hepatocytes [84], as well as neutrophils from patients with alcoholic liver disease [85], suggesting a potential role of it in liver diseases.

Yu et al. [84] reported that miR-223 expression was increased in mouse livers after 75 min of ischemia followed by 120 min of reperfusion when compared to control mouse livers. Upregulated miR-223 expression was positively correlated with serum AST and ALT activities, which were the most important serum markers of liver injury. Schueller et al. [85] reported that the miR-223 level is upregulated in liver and serum from mice after experimental acute liver injury, as well as from patients with acute liver failure, and its level is correlated with the degree of liver injury and hepatocyte death. However, Van Caster et al. [76] confirmed the elevation of miR-223 in the liver after IRI, but they found no correlation between its level and serum AST/ALT activities. Moreover, circulating the miR-223 level remained unchanged in the same study [76]. Overall, an increase in hepatic miR-223 expression is associated with liver IRI, but its effects and underlying mechanism(s) in liver IRI still remain unclear.

### 5.4. miR-370 and Liver IRI

Li et al. [86] reported that miR-370 expression was significantly increased in the liver after IRI, and the ischemic preconditioning (IPC) inhibited the upregulation of miR-370 with the amelioration of liver IRI. The authors further confirmed that transforming growth factor-β receptor II (TβRII) is a direct target gene of miR-370. In IRI mouse livers, TβRII was downregulated. Inhibition of miR-370 restored the expression of TβRII and its downstream target phosphorylated mothers against decapentaplegic homolog 3 (Smad3), and ameliorated hepatic IRI. Another study [87] confirmed the increase of miR-370 in IRI mouse livers. miR-370 also activates NF-κB pathway to exaggerate liver IRI [87]. Collectively, miR-370 exaggerates liver IRI by inhibiting TβRII pathway and activating the NF-κB pathway.

### 5.5. miR-155 and liver IRI

miR-155 expression is induced by various inflammatory mediators, and is associated with both innate and adaptive immune responses [88]. Tang, B. et al. [88] demonstrated that miR-155 deficiency protected mouse livers from IRI using a liver transplantation model in cold temperature. The authors provided evidence that miR-155 deficiency upregulated the expression of suppressors of cytokine signaling1 (SOCS1), a target gene of miR-155, to promote M2 macrophages development, suppress Th17 cell differentiation, and repress IL-17 expression. Beyond the protective roles in this cold ischemic injury, Li et al. [89] reported that miR-155 deficiency also protected the liver from warm IRI using a partial liver IRI model. The authors found that miR-155 deficiency inhibited the activation of Kupffer cells and the expression of inflammatory cytokines during liver IRI. Collectively, miR-155 deficiency protects the liver against IRI by inhibiting macrophage activation and inflammation in the livers.

### 5.6. Other miRNAs and Liver IRI

In addition to the miRNAs discussed above, several other miRNAs are also involved in liver IRI. Ng et al. [63] reported that circulating miR-148a and miR-1246 levels were potential biomarkers in predicting HCC recurrence after liver transplantation in the early phase. Zheng et al. [90] reported that miR-148a expression was increased in the livers after IRI with a negative correlation with calcium/calmodulin-dependent protein kinase type II alpha chain (CaMKIIα). The authors proposed that the inhibition of CaMKIIα might be the main protective mechanism of miR-148a on liver IRI. miR-146a was downregulated in the early stage of liver IRI in a rat model [91]. miR-124 is downregulated after hepatic IRI, and the H_2_O_2_-induced apoptosis of human hepatic L02 cells was significantly inhibited by miR-124 activation, which repressed the Ras-related protein Rab-38 (Rab38) gene and activated the Akt survival pathway [92]. The miR-30b [93] level was downregulated after hepatic IRI, and miR-30b activation alleviated hepatic IRI by inhibiting autophagy. The miR-17 [94] level was increased after hepatic IRI. Additionally, miR-17 repressed signal transductions and activation of transcription-3 (Stat3) activation to exaggerate liver IRI. miR-200c [95] may mediate the protective effects of sevoflurane on hepatic IRI. miR-182-5p [96] inhibits proinflammatory cytokine production in LPS-treated macrophages and attenuates liver IRI by inactivating toll-like receptor 4 (TLR4). Mitogen-activated protein kinase 6 (MAPK6) is a target of miR-133a-5p [97], and miR-133a-5p/MAPK6 axis is involved with propofol’s protective effects in hepatic IRI in vivo and hypoxia/reoxygenation (H/R) injury in vitro. miR-1224 expression [98] was upregulated in animal hepatocytes after IRI in vivo and in vitro, and increased in the livers and serum of patients with acute liver failure (ALF). miR-1224 inhibited hepatocyte proliferation and survival in acute liver failure by targeting the antiapoptotic gene nuclear factor I/B (NFIB). Moreover, deregulated expression of miR-410-3p, miR-490-3p, and miR-582-5p [99] was also associated with liver IRI.

Overall, many miRNAs are involved in liver IRI. The expression change of certain important miRNAs discussed above in circulation and liver, and their effects and main mechanisms in liver IRI had also been summarized in Table 1.

## 6. LncRNAs and Liver IRI

LncRNAs are the majority of noncoding RNAs which are also widely involved in many physiological and pathophysiological processes by functioning as *cis*-tether, cistargeting, *trans*-targeting, enhancer, decoy, scaffold, allosteric modification, co-activator, or corepressor to modulate gene expression in various cell types [100].

More recently, there had been evidence that LncRNAs may also play important roles in liver injury. Su et al. [101] reported that LncRNA TUG1 was downregulated in mouse livers after cold storage, which is a major problem affecting liver transplantation. TUG1 overexpression attenuated cold storage-induced apoptosis, inflammation, and oxidative stress of hepatocytes in vivo and in vitro, suggesting a potential role of TUG1 as a therapeutic target for the prevention of cold-induced liver injury in hepatic transplantation. For the first time, we had determined the LncRNA profile in mouse plasma after liver IRI using microarray technology [100]. Sixty-four LncRNAs were upregulated, and 244 LncRNAs were downregulated in the plasma of mice after an hour of 70% hepatic ischemia and 6 h of reperfusion. These findings suggested that certain plasma LncRNAs have the potential of becoming novel biomarkers for hepatic IRI.

We also determined that the LncRNA expression profile in mouse livers after IRI [102]. In a liver IRI mouse model with 1-h ischemia and 6-h reperfusion, 71 LncRNAs were identified with upregulation and 27 LncRNAs with downregulation. LncRNA AK139328 exhibited the highest expression level in normal mouse livers among the upregulated LncRNAs. Inhibition of the hepatic AK139328 resulted in decreased plasma aminotransferase activities and reduced necrosis area in the livers with a decrease in caspase-3 activation after liver IRI. Silencing of AK139328 also activated Akt, and repressed NF-κB and inflammation in mouse livers after IRI [102]. Moreover, LncRNA AK143693, which was downregulated in mouse liver after IRI [102], also modulated Akt activity in hepatocytes. AK143693 was also renamed as LncRNA Suppressor of Hepatic Gluconeogenesis and Lipogenesis (LncSHGL) because it suppresses gluconeogenesis and lipogenesis of hepatocytes in vitro and in vivo. AK143693 recruits heterogeneous nuclear ribonucleoprotein A1 (hnRNPA1) to enhance calmodulin (CaM) mRNAs translation, elevating CaM protein to activate Akt independent of insulin and calcium, finally leading to the suppression of gluconeogenic and lipogenic pathways in hepatocytes [103]. Clearly, an increase in AK139328 expression plus a decrease in AK143693 (LncSHGL) expression together exerts deleterious effects on liver IRI by impairing Akt activity in hepatocytes. These findings suggested that LncRNA AK139328 and AK143693 might be novel therapeutic targets for liver IRI.

## 7. Summary and Perspective

Hepatic IRI is a severe clinical issue affecting millions of people worldwide. Ischemic preconditioning and ischemic postconditioning could be effective strategies for attenuating liver IRI in clinics. Moreover, antioxidants, scavengers of free radicals, and inflammatory inhibitors could also be used to protect hepatocytes against apoptosis and necrosis when IRI happens. In the past decade, intensive studies had shed light on the mechanisms of liver IRI and provided many potential diagnostic biomarkers and therapeutical targets. Inhibition of certain miRNA, such as miR-122, modulation of certain LncRNAs, such as AK143693 (LncSHGL) and AK139328, and activation of the PPARγ-*FAM3A* axis might be potential strategies for treating liver IRI. In particular, oral administration of PPARγ agonists before liver surgery or liver transplantation to activate hepatic *FAM3A* pathways holds great promise for reducing liver IRI in clinics (Figure 2).

## Figures and Tables

**Table 1:** Summarization of the roles and mechanisms of certain important miRNAs in liver IRI.

miRNAs	Change in IRI	Effects	Proposed Mechanism
miR-34a	Increased in liver and circulation	Exaggerating liver IRI	Suppressing Nrf2 and SIRT1 pathways
miR-122	Increased in liver and circulation	Exaggerating liver IRI	Inhibiting IGF-1R/Akt pathway
miR-155	Increased in liver and circulation	Exaggerating liver IRI	Activating Kupffer cells and promoting inflammation
miR-223	Increased in liver and circulation	Exaggerating liver IR	Unclear
miR-370	Increased in liver and circulation	Exaggerating liver IRI	Inhibiting TβRII pathway and activating NF-κB pathway

The corresponding references for each miRNA are indicated in the context.

## References

[1] Saidi R.F., Kenari S.K. (2014). Liver ischemia/reperfusion injury: An overview. J. Investig. Surg..

[2] Nastos C., Kalimeris K., Papoutsidakis N., Tasoulis M.K., Lykoudis P.M., Theodoraki K., Nastou D., Smyrniotis V., Arkadopoulos N. (2014). Global consequences of liver ischemia/reperfusion injury. Oxid. Med. Cell. Longev..

[3] Cheng G., Palanisamy A.P., Evans Z.P., Sutter A.G., Jin L., Singh I., May H., Schmidt M.G., Chavin K.D. (2013). Cerulenin blockade of fatty acid synthase reverses hepatic steatosis in ob/ob mice. PLoS ONE.

[4] Covington S.M., Bauler L.D., Toledo-Pereyra L.H. (2016). Akt: A Therapeutic Target in Hepatic Ischemia–Reperfusion Injury. J. Investig. Surg..

[5] Quillin R.C., Guarrera J.V. (2018). Hypothermic machine perfusion in liver transplantation. Liver Transpl..

[6] Orci L.A., Lacotte S., Delaune V., Slits F., Oldani G., Lazarevic V., Rossetti C., Rubbia-Brandt L., Morel P., Toso C. (2018). Effects of the gut-liver axis on ischemia-mediated hepatocellular carcinoma recurrence in the mouse liver. J. Hepatol..

[7] Liu Y.I., Liu Z., Chen Y., Xu K., Dong J. (2016). PPARgamma activation reduces ischemia/reperfusion-induced metastasis in a murine model of hepatocellular carcinoma. Exp. Ther. Med..

[8] Huang Y., Rabb H., Womer K.L. (2007). Ischemia-reperfusion and immediate T cell responses. Cell Immunol..

[9] Gracia-Sancho J., Casillas-Ramirez A., Peralta C. (2015). Molecular pathways in protecting the liver from ischaemia/reperfusion injury: A 2015 update. Clin. Sci..

[10] Ma Z., Xin Z., Di W., Yan X., Li X., Reiter R.J., Yang Y. (2017). Melatonin and mitochondrial function during ischemia/reperfusion injury. Cell. Mol. Life Sci..

[11] Zhang M., Carroll M.C. (2007). Natural IgM-mediated innate autoimmunity: A new target for early intervention of ischemia-reperfusion injury. Expert Opin. Biol. Ther..

[12] Cannistrà M., Ruggiero M., Zullo A., Gallelli G., Serafini S., Maria M., Naso A., Grande R., Serra R., Nardo B. (2016). Original research: Hepatic ischemia reperfusion injury: A systematic review of literature and the role of current drugs and biomarkers. Int. J. Surg..

[13] Jia J.J., Li J.H., Jiang L., Lin B.Y., Wang L., Su R., Zhou L., Zheng S.S. (2015). Liver protection strategies in liver transplantation. Hepatobiliary Pancreat. Dis. Int..

[14] Jeong J.S., Kim D., Kim K.Y., Ryu S., Han S., Shin B.S., Kim G.S., Gwak M.S., Ko J.S. (2017). Ischemic preconditioning produces comparable protection against hepatic ischemia/reperfusion injury under isoflurane and sevoflurane anesthesia in rats. Transplant. Proc..

[15] De Almeida T.N., Victorino J.P., Bistafa Liu J., Tofoli Queiroz Campos D., Graf C., Jordani M.C., Carneiro D’albuquerque L.A., Mendes K.D.S., Castro E.S.O. (2018). Effect of hepatic preconditioning with the use of methylene blue on the liver of wistar rats submitted to ischemia and reperfusion. Transplant. Proc..

[16] Pontes H.B.D., Pontes J., Azevedo Neto E., Vendas G., Miranda J.V.C., Dias L., Oliva J., Almeida M.H.M., Chaves I.O., Sampaio T.L. (2018). Evaluation of the effects of atorvastatin and ischemic postconditioning preventing on the ischemia and reperfusion injury: experimental study in rats. Braz. J. Cardiovasc. Surg..

[17] Alva N., Bardallo R.G., Basanta D., Palomeque J., Carbonell T. (2018). Preconditioning-like properties of short-term hypothermia in isolated perfused rat liver (IPRL) system. Int. J. Mol. Sci..

[18] Camara-Lemarroy C.R., Guzman-de la Garza F.J., Alarcon-Galvan G., Cordero-Perez P., Munoz-Espinosa L., Torres-Gonzalez L., Fernandez-Garza N.E. (2014). Hepatic ischemia/reperfusion injury is diminished by atorvastatin in Wistar rats. Arch Med. Res..

[19] Theodoraki K., Karmaniolou I., Tympa A., Tasoulis M.K., Nastos C., Vassiliou I., Arkadopoulos N., Smyrniotis V. (2016). Beyond preconditioning: postconditioning as an alternative technique in the prevention of liver ischemia-reperfusion injury. Oxid. Med. Cell. Longev..

[20] Schlegel A.A., Kalisvaart M., Muiesan P. (2018). Machine perfusion in liver transplantation: An essential treatment or just an expensive toy?. Minerva Anestesiol..

[21] Donato M., Evelson P., Gelpi R.J. (2017). Protecting the heart from ischemia/reperfusion injury: An update on remote ischemic preconditioning and postconditioning. Curr. Opin. Cardiol..

[22] Lin H.C., Liu S.Y., Yen E.Y., Li T.K., Lai I.R. (2017). microRNA-183 mediates protective postconditioning of the liver by repressing Apaf-1. Antioxid. Redox Signal..

[23] Feyzizadeh S., Badalzadeh R. (2017). Application of ischemic postconditioning’s algorithms in tissues protection: Response to methodological gaps in preclinical and clinical studies. J. Cell. Mol. Med..

[24] Lehrke M., Lazar M.A. (2005). The many faces of PPARgamma. Cell.

[25] Balakumar P., Rose M., Singh M. (2007). PPAR Ligands: Are they potential agents for cardiovascular disorders?. Pharmacology.

[26] Ruan X., Zheng F., Guan Y. (2008). PPARs and the kidney in metabolic syndrome. Am. J. Physiol. Ren. Physiol..

[27] Yang J., Zhang D., Li J., Zhang X., Fan F., Guan Y. (2009). Role of PPARgamma in renoprotection in Type 2 diabetes: Molecular mechanisms and therapeutic potential. Clin. Sci..

[28] Gross B., Pawlak M., Lefebvre P., Staels B. (2017). PPARs in obesity-induced T2DM, dyslipidaemia and NAFLD. Nat. Rev. Endocrinol..

[29] Janani C., Ranjitha Kumari B.D. (2015). PPAR gamma gene—A review. Diabetes Metab. Syndr..

[30] Marion-Letellier R., Savoye G., Ghosh S. (2016). Fatty acids, eicosanoids and PPAR gamma. Eur. J. Pharmacol..

[31] Casillas-Ramirez A., Amine-Zaouali M., Massip-Salcedo M., Padrissa-Altes S., Bintanel-Morcillo M., Ramalho F., Serafin A., Rimola A., Arroyo V., Rodes J. (2008). Inhibition of angiotensin II action protects rat steatotic livers against ischemia-reperfusion injury. Crit. Care Med..

[32] Kuboki S., Shin T., Huber N., Eismann T., Galloway E., Schuster R., Blanchard J., Zingarelli B., Lentsch A.B. (2008). Peroxisome proliferator-activated receptor-gamma protects against hepatic ischemia/reperfusion injury in mice. Hepatology.

[33] Shin T., Kuboki S., Huber N., Eismann T., Galloway E., Schuster R., Blanchard J., Pritts T.A., Lentsch A.B. (2008). Activation of peroxisome proliferator-activated receptor-gamma during hepatic ischemia is age-dependent. J. Surg. Res..

[34] Xu Y., Yao J., Zou C., Zhang H., Zhang S., Liu J., Ma G., Jiang P., Zhang W. (2017). Asiatic acid protects against hepatic ischemia/reperfusion injury by inactivation of Kupffer cells via PPARgamma/NLRP3 inflammasome signaling pathway. Oncotarget.

[35] Chen K., Li J.J., Li S.N., Feng J., Liu T., Wang F., Dai W.Q., Xia Y.J., Lu J., Zhou Y.Q. (2017). 15-Deoxy-Delta(12,14)-prostaglandin J2 alleviates hepatic ischemia-reperfusion injury in mice via inducing antioxidant response and inhibiting apoptosis and autophagy. Acta Pharmacol. Sin..

[36] Ruan W., Liu Q., Chen C., Li S., Xu J. (2016). Limb remote ischemic preconditioning attenuates liver ischemia reperfusion injury by activating autophagy via modulating PPAR-gamma pathway. Zhong Nan Da Xue Xue Bao Yi Xue Ban.

[37] Koh E.J., Yoon S.J., Lee S.M. (2013). Losartan protects liver against ischaemia/reperfusion injury through PPAR-gamma activation and receptor for advanced glycation end-products down-regulation. Br. J. Pharmacol..

[38] Matsuda A., Jacob A., Wu R., Zhou M., Aziz M., Wang P. (2013). Milk fat globule—EGF factor VIII ameliorates liver injury after hepatic ischemia-reperfusion. J. Surg. Res..

[39] Zhai D., Zhang J., Zheng Q., Li Z., Zhang J., Tian Y. (2008). Significance of rosiglitazone inhibiting TLR4 expression in partial hepatic ischemia/reperfusion of mice. J. Huazhong Univ. Sci. Technol. Med. Sci..

[40] Casillas-Ramirez A., Zaouali A., Padrissa-Altes S., Ben Mosbah I., Pertosa A., Alfany-Fernandez I., Bintanel-Morcillo M., Xaus C., Rimola A., Rodes J. (2009). Insulin-like growth factor and epidermal growth factor treatment: New approaches to protecting steatotic livers against ischemia-reperfusion injury. Endocrinology.

[41] Casillas-Ramirez A., Alfany-Fernandez I., Massip-Salcedo M., Juan M.E., Planas J.M., Serafin A., Pallas M., Rimola A., Rodes J., Peralta C. (2011). Retinol-binding protein 4 and peroxisome proliferator-activated receptor-gamma in steatotic liver transplantation. J. Pharmacol. Exp. Ther..

[42] Jimenez-Castro M.B., Elias-Miro M., Mendes-Braz M., Lemoine A., Rimola A., Rodes J., Casillas-Ramirez A., Peralta C. (2012). Tauroursodeoxycholic acid affects PPARgamma and TLR4 in Steatotic liver transplantation. Am. J. Transplant..

[43] Zaouali M.A., Padrissa-Altes S., Ben Mosbah I., Alfany-Fernandez I., Massip-Salcedo M., Casillas-Ramirez A., Bintanel-Morcillo M., Boillot O., Serafin A., Rimola A. (2010). Improved rat steatotic and nonsteatotic liver preservation by the addition of epidermal growth factor and insulin-like growth factor-I to University of Wisconsin solution. Liver Transpl..

[44] Somi M.H., Hajipour B., Asl N.A., Estakhri R., Azar A.N., Zade M.N., Haghjou A.G., Vatankhah A.M. (2009). Pioglitazone attenuates ischemia/reperfusion-induced liver injury in rats. Transplant. Proc..

[45] Elias-Miro M., Jimenez-Castro M.B., Mendes-Braz M., Casillas-Ramirez A., Peralta C. (2012). The Current Knowledge of the Role of PPAR in Hepatic Ischemia-Reperfusion Injury. PPAR Res..

[46] Zhou Y., Jia S., Wang C., Chen Z., Chi Y., Li J., Xu G., Guan Y., Yang J. (2013). FAM3A is a target gene of peroxisome proliferator-activated receptor gamma. Biochim. Biophys. Acta.

[47] Wang C., Chi Y., Li J., Miao Y., Li S., Su W., Jia S., Chen Z., Du S., Zhang X. (2014). FAM3A activates PI3K p110alpha/Akt signaling to ameliorate hepatic gluconeogenesis and lipogenesis. Hepatology.

[48] Yang W., Wang J., Chen Z., Chen J., Meng Y., Chen L., Chang Y., Geng B., Sun L., Dou L. (2017). NFE2 Induces miR-423-5p to Promote Gluconeogenesis and Hyperglycemia by Repressing the Hepatic FAM3A-ATP-Akt Pathway. Diabetes.

[49] Chi Y., Li J., Li N., Chen Z., Ma L., Peng W., Pan X., Li M., Yu W., He X. (2017). FAM3A enhances adipogenesis of 3T3-L1 preadipocytes via activation of ATP-P2 receptor-Akt signaling pathway. Oncotarget.

[50] Jia S., Chen Z., Li J., Chi Y., Wang J., Li S., Luo Y., Geng B., Wang C., Cui Q. (2014). FAM3A promotes vascular smooth muscle cell proliferation and migration and exacerbates neointima formation in rat artery after balloon injury. J. Mol. Cell. Cardiol..

[51] Carini R., Grazia De Cesaris M., Splendore R., Baldanzi G., Nitti M.P., Alchera E., Filigheddu N., Domenicotti C., Pronzato M.A., Graziani A. (2004). Role of phosphatidylinositol 3-kinase in the development of hepatocyte preconditioning. Gastroenterology.

[52] Dal Ponte C., Alchera E., Follenzi A., Imarisio C., Prat M., Albano E., Carini R. (2011). Pharmacological postconditioning protects against hepatic ischemia/reperfusion injury. Liver Transpl..

[53] Ke B., Shen X.D., Ji H., Kamo N., Gao F., Freitas M.C., Busuttil R.W., Kupiec-Weglinski J.W. (2012). HO-1-STAT3 axis in mouse liver ischemia/reperfusion injury: Regulation of TLR4 innate responses through PI3K/PTEN signaling. J. Hepatol..

[54] Kamo N., Ke B., Busuttil R.W., Kupiec-Weglinski J.W. (2013). PTEN-mediated Akt/beta-catenin/Foxo1 signaling regulates innate immune responses in mouse liver ischemia/reperfusion injury. Hepatology.

[55] Chen Z., Wang J., Yang W., Chen J., Meng Y., Geng B., Cui Q., Yang J. (2017). FAM3A mediates PPARγ’s protection in liver ischemia-reperfusion injury by activating Akt survival pathway and repressing inflammation and oxidative stress. Oncotarget.

[56] Lentsch A.B., Kato A., Yoshidome H., McMasters K.M., Edwards M.J. (2000). Inflammatory mechanisms and therapeutic strategies for warm hepatic ischemia/reperfusion injury. Hepatology.

[57] Song Q., Gou W.L., Zhang R. (2015). FAM3A Protects HT22 Cells Against Hydrogen Peroxide-Induced Oxidative Stress Through Activation of PI3K/Akt but not MEK/ERK Pathway. Cell. Physiol. Biochem..

[58] Song Q., Gou W.L., Zhang R. (2016). FAM3A attenuates ER stress-induced mitochondrial dysfunction and apoptosis via CHOP-Wnt pathway. Neurochem. Int..

[59] Kapranov P., Cheng J., Dike S., Nix D.A., Duttagupta R., Willingham A.T., Stadler P.F., Hertel J., Hackermuller J., Hofacker I.L. (2007). RNA maps reveal new RNA classes and a possible function for pervasive transcription. Science.

[60] Alvarez-Garcia I., Miska E.A. (2005). MicroRNA functions in animal development and human disease. Development.

[61] Zhang B., Farwell M.A. (2008). microRNAs: A new emerging class of players for disease diagnostics and gene therapy. J. Cell. Mol. Med..

[62] Schickel R., Boyerinas B., Park S.M., Peter M.E. (2008). MicroRNAs: Key players in the immune system, differentiation, tumorigenesis and cell death. Oncogene.

[63] Ng K.T., Lo C.M., Wong N., Li C.X., Qi X., Liu X.B., Geng W., Yeung O.W., Ma Y.Y., Chan S.C. (2016). Early-phase circulating miRNAs predict tumor recurrence and survival of hepatocellular carcinoma patients after liver transplantation. Oncotarget.

[64] Zheng W., Men H., Li J., Xing Y., Wu B., Wang Z., Li J., Teng D., Shi Y., Li J. (2016). Global MicroRNA Expression Profiling of Mouse Livers following Ischemia-Reperfusion Injury at Different Stages. PLoS ONE.

[65] Morita T., Ishikawa M., Sakamoto A. (2015). Identical MicroRNAs Regulate Liver Protection during Anaesthetic and Ischemic Preconditioning in Rats: An animal study. PLoS ONE.

[66] Xu C.F., Yu C.H., Li Y.M. (2009). Regulation of hepatic microRNA expression in response to ischemic preconditioning following ischemia/reperfusion injury in mice. OMICS J. Integr. Biol..

[67] Gehrau R.C., Mas V.R., Dumur C.I., Ladie D.E., Suh J.L., Luebbert S., Maluf D.G. (2013). Regulation of molecular pathways in ischemia-reperfusion injury after liver transplantation. Transplantation.

[68] Lagos-Quintana M., Rauhut R., Yalcin A., Meyer J., Lendeckel W., Tuschl T. (2002). Identification of tissue-specific microRNAs from mouse. Curr. Biol..

[69] Chang J., Nicolas E., Marks D., Sander C., Lerro A., Buendia M.A., Xu C., Mason W.S., Moloshok T., Bort R. (2004). miR-122, a mammalian liver-specific microRNA, is processed from hcr mRNA and may downregulate the high affinity cationic amino acid transporter CAT-1. RNA Biol..

[70] Hu J., Xu Y., Hao J., Wang S., Li C., Meng S. (2012). MiR-122 in hepatic function and liver diseases. Protein Cell.

[71] Tsai W.C., Hsu S.D., Hsu C.S., Lai T.C., Chen S.J., Shen R., Huang Y., Chen H.C., Lee C.H., Tsai T.F. (2012). MicroRNA-122 plays a critical role in liver homeostasis and hepatocarcinogenesis. J. Clin. Investig..

[72] Wen J., Friedman J.R. (2012). miR-122 regulates hepatic lipid metabolism and tumor suppression. J. Clin. Investig..

[73] Andersson P., Gidlof O., Braun O.O., Gotberg M., van der Pals J., Olde B., Erlinge D. (2012). Plasma levels of liver-specific miR-122 is massively increased in a porcine cardiogenic shock model and attenuated by hypothermia. Shock.

[74] Roderburg C., Benz F., Vargas Cardenas D., Koch A., Janssen J., Vucur M., Gautheron J., Schneider A.T., Koppe C., Kreggenwinkel K. (2015). Elevated miR-122 serum levels are an independent marker of liver injury in inflammatory diseases. Liver Int..

[75] Yang M., Antoine D.J., Weemhoff J.L., Jenkins R.E., Farhood A., Park B.K., Jaeschke H. (2014). Biomarkers distinguish apoptotic and necrotic cell death during hepatic ischemia/reperfusion injury in mice. Liver Transpl..

[76] Van Caster P., Brandenburger T., Strahl T., Metzger S., Bauer I., Pannen B., Braun S. (2015). Circulating microRNA-122, -21 and -223 as potential markers of liver injury following warm ischaemia and reperfusion in rats. Mol. Med. Rep..

[77] Selten J.W., Verhoeven C.J., Heedfeld V., Roest H.P., de Jonge J., Pirenne J., van Pelt J., Ijzermans J.N.M., Monbaliu D., van der Laan L.J.W. (2017). The release of microRNA-122 during liver preservation is associated with early allograft dysfunction and graft survival after transplantation. Liver Transpl..

[78] Xiao Q., Ye Q.F., Wang W., Fu B.Q., Xia Z.P., Liu Z.Z., Zhang X.J., Wang Y.F. (2017). Mild hypothermia pretreatment protects hepatocytes against ischemia reperfusion injury via down-regulating miR-122 and IGF-1R/AKT pathway. Cryobiology.

[79] Akbari G., Mard S.A., Dianat M., Mansouri E. (2017). The hepatoprotective and microRNAs downregulatory effects of crocin following hepatic ischemia-reperfusion injury in rats. Oxid. Med. Cell. Longev..

[80] Mard S.A., Akbari G., Dianat M., Mansouri E. (2017). Protective effects of crocin and zinc sulfate on hepatic ischemia-reperfusion injury in rats: A comparative experimental model study. Biomed. Pharmacother..

[81] Huang X., Gao Y., Qin J., Lu S. (2014). The role of miR-34a in the hepatoprotective effect of hydrogen sulfide on ischemia/reperfusion injury in young and old rats. PLoS ONE.

[82] Kim H.J., Joe Y., Yu J.K., Chen Y., Jeong S.O., Mani N., Cho G.J., Pae H.O., Ryter S.W., Chung H.T. (2015). Carbon monoxide protects against hepatic ischemia/reperfusion injury by modulating the miR-34a/SIRT1 pathway. Biochim. Biophys. Acta.

[83] Shan W., Gao L., Zeng W., Hu Y., Wang G., Li M., Zhou J., Ma X., Tian X., Yao J. (2015). Activation of the SIRT1/p66shc antiapoptosis pathway via carnosic acid-induced inhibition of miR-34a protects rats against nonalcoholic fatty liver disease. Cell Death Dis..

[84] Yu C.H., Xu C.F., Li Y.M. (2009). Association of MicroRNA-223 expression with hepatic ischemia/reperfusion injury in mice. Dig. Dis. Sci..

[85] Schueller F., Roy S., Loosen S.H., Alder J., Koppe C., Schneider A.T., Wandrer F., Bantel H., Vucur M., Mi Q.S. (2017). miR-223 represents a biomarker in acute and chronic liver injury. Clin. Sci..

[86] Li L., Li G., Yu C., Shen Z., Xu C., Feng Z., Zhang X., Li Y. (2015). A role of microRNA-370 in hepatic ischaemia-reperfusion injury by targeting transforming growth factor-beta receptor II. Liver Int..

[87] Zhu J., Zhu F., Song W., Zhang B., Zhang X., Jin X., Li H. (2017). Altered miR-370 expression in hepatic ischemia-reperfusion injury correlates with the level of nuclear kappa B (NF-kappaB) related factors. Gene.

[88] Tang B., Wang Z., Qi G., Yuan S., Yu S., Li B., Wei Y., Huang Q., Zhai R., He S. (2015). MicroRNA-155 deficiency attenuates ischemia-reperfusion injury after liver transplantation in mice. Transpl. Int..

[89] Li Y., Ma D., Wang Z., Yang J. (2017). MicroRNA-155 deficiency in Kupffer cells ameliorates liver ischemia-reperfusion injury in mice. Transplantation.

[90] Zheng D., He D., Lu X., Sun C., Luo Q., Wu Z. (2016). The miR-148a alleviates hepatic ischemia/reperfusion injury in mice via targeting CaMKIIalpha. Xi Bao Yu Fen Zi Mian Yi Xue Za Zhi.

[91] Chen Q., Kong L., Xu X., Geng Q., Tang W., Jiang W. (2013). Down-regulation of microRNA-146a in the early stage of liver ischemia-reperfusion injury. Transplant. Proc..

[92] Li X., Yi S., Deng Y., Cheng J., Wu X., Liu W., Tai Y., Chen G., Zhang Q., Yang Y. (2014). MiR-124 protects human hepatic L02 cells from H2O2-induced apoptosis by targeting Rab38 gene. Biochem. Biophys. Res. Commun..

[93] Li S.P., He J.D., Wang Z., Yu Y., Fu S.Y., Zhang H.M., Zhang J.J., Shen Z.Y. (2016). miR-30b inhibits autophagy to alleviate hepatic ischemia-reperfusion injury via decreasing the Atg12-Atg5 conjugate. World J. Gastroenterol..

[94] Li S., Zhang J., Wang Z., Wang T., Yu Y., He J., Zhang H., Yang T., Shen Z. (2016). MicroRNA-17 regulates autophagy to promote hepatic ischemia/reperfusion injury via suppression of signal transductions and activation of transcription-3 expression. Liver Transpl..

[95] Wu Y., Gu C., Huang X. (2016). Sevoflurane protects against hepatic ischemia/reperfusion injury by modulating microRNA-200c regulation in mice. Biomed. Pharmacother..

[96] Jiang W., Liu G., Tang W. (2016). MicroRNA-182-5p Ameliorates Liver Ischemia-Reperfusion Injury by Suppressing Toll-Like Receptor 4. Transplant. Proc..

[97] Hao W., Zhao Z.H., Meng Q.T., Tie M.E., Lei S.Q., Xia Z.Y. (2017). Propofol protects against hepatic ischemia/reperfusion injury via miR-133a-5p regulating the expression of MAPK6. Cell Biol. Int..

[98] Roy S., Bantel H., Wandrer F., Schneider A.T., Gautheron J., Vucur M., Tacke F., Trautwein C., Luedde T., Roderburg C. (2017). miR-1224 inhibits cell proliferation in acute liver failure by targeting the antiapoptotic gene Nfib. J. Hepatol..

[99] Zhang C., Huang J., An W. (2017). Hepatic stimulator substance resists hepatic ischemia/reperfusion injury by regulating Drp1 translocation and activation. Hepatology.

[100] Chen Z., Luo Y., Yang W., Ding L., Wang J., Tu J., Geng B., Cui Q., Yang J. (2015). Comparison Analysis of Dysregulated LncRNA Profile in Mouse Plasma and Liver after Hepatic Ischemia/Reperfusion Injury. PLoS ONE.

[101] Su S., Liu J., He K., Zhang M., Feng C., Peng F., Li B., Xia X. (2016). Overexpression of the long noncoding RNA TUG1 protects against cold-induced injury of mouse livers by inhibiting apoptosis and inflammation. FEBS J..

[102] Chen Z., Jia S., Li D., Cai J., Tu J., Geng B., Guan Y., Cui Q., Yang J. (2013). Silencing of long noncoding RNA AK139328 attenuates ischemia/reperfusion injury in mouse livers. PLoS ONE.

[103] Wang J., Yang W., Chen Z., Chen J., Meng Y., Feng B., Sun L., Dou L., Li J., Cui Q. (2018). Long Non-coding RNA LncSHGL recruits hnRNPA1 to suppress hepatic gluconeogenesis and lipogenesis. Diabetes.

